# Antioxidant Capacity, Inflammatory Response, Carcass Characteristics and Meat Quality of Hu Sheep in Response to Dietary Soluble Protein Levels with Decreased Crude Protein Content

**DOI:** 10.3390/antiox12122098

**Published:** 2023-12-11

**Authors:** Xin Zhang, Zhenbin Zhang, Yiquan Sun, Yang Liu, Xinhuang Zhong, Jun Zhu, Xiang Yu, Yue Lu, Zhiqi Lu, Xuezhao Sun, Huanyong Han, Mengzhi Wang

**Affiliations:** 1 State Key Laboratory of Sheep Genetic Improvement and Healthy Production, Xinjiang Academy of Agricultural Reclamation Sciences, Shihezi 832000, China; 2 Laboratory of Metabolic Manipulation of Herbivorous Animal Nutrition, College of Animal Science and Technology, Yangzhou University, Yangzhou 225009, China; 3 AgResearch Limited, Grasslands Research Centre, Palmerston North 4410, New Zealand; xuezhao.sun@agresearch.co.nz

**Keywords:** soluble protein, low-protein diets, antioxidant capacity, inflammatory response, carcass characteristics, meat quality

## Abstract

Manipulating dietary nutrients, especially protein fractions, holds significance in enhancing the antioxidant capacity and immunity function of ruminants. This study investigated the impact of dietary adjustments in soluble protein (SP) levels, in conjunction with a reduction in crude protein (CP) content, on the antioxidant capacity, inflammatory response, carcass characteristics, and meat quality of sheep. This study had four dietary treatments, including a control diet (CON) adhering to NRC standards with a CP content of 16.7% on a dry matter basis and three diets with an approximately 10% reduction in CP content compared to CON with SP levels (% of CP) of 21.2 (SPA), 25.9 (SPB) and 29.4% (SPC), respectively. Thirty-two healthy male Hu sheep, with an initial live weight of 40.37 ± 1.18 kg and age of 6 months, were randomly divided into four groups to receive these respective diets. Our data revealed no significant differences in slaughter performance among treatments (*p* > 0.05), although low-protein treatments decreased the stomachus compositus index (*p* < 0.05). Compared with CON, as SP was adjusted to 21.2%, total antioxidant capacity (T-AOC) and catalase (CAT) concentrations were decreased in the serum (*p* < 0.05), glutathione peroxidase (GSH-Px) content was decreased in jejunum and ileum (*p* < 0.05), superoxide dismutase (SOD) concentration was reduced in the duodenum (*p* < 0.05), and malondialdehyde (MDA) content was increased in spleen and ileum (*p* < 0.05). On the other hand, pro-inflammatory cytokine (IL-1β, IL-6 and IL-8) contents were upregulated in the serum (*p* < 0.05), while immunoglobulin (IgA and IgM) contents were reduced in the duodenum (*p* < 0.05) with SP adjustments. Additionally, the SPB and SPC diets reduced the content of saturated fatty acids and increased the content of polyunsaturated fatty acids compared with CON (*p* < 0.05), along with retention in the tenderness and water-holding capacity of the *longissimus lumborum* muscle. In summary, reducing CP by 10% with an SP proportion of ~25–30% improved meat quality without compromising antioxidant capacity and immunity function, while lower SP levels had adverse effects.

## 1. Introduction

With the rapid growth of human demand for high-quality animal-derived proteins, such as beef, mutton and dairy products, the breeding volume of livestock, particularly ruminants, is continuously expanding [1]. However, the intensity of greenhouse gas emissions (e.g., methane and nitrous oxide) from ruminants is higher than that of monogastric livestock animals [2]. Therefore, developing sustainable ruminant production systems that prioritize environmental protection, resource conservation, and high product quality has become an inevitable choice.

Dietary nutrient manipulations, such as intervening protein fractions levels, have significant implications for nutrient utilization in ruminants, as well as lowering their environmental footprint in terms of nitrogen (N) emissions—for instance, reducing dietary N levels or adjusting the proportion of rumen-degradable proteins [3,4]. Our previous in vitro study elucidated the responses of bacterial and protozoal communities to different soluble protein (SP) levels and the role of microbial interactions on N metabolism. The results indicated that regulating SP levels (% of CP) to 30% or 40% could modify bacterial and protozoal communities to enhance N utilization efficiency [5]. Furthermore, we found that altering dietary SP levels (approximately 25–30%) with a decrease in crude protein (CP) content could improve N efficiency by regulating the rumen microbiome and metabolites in vivo [6]. While our previous studies mainly focused on N metabolism and its potential to reduce the environmental footprint, farmers and consumers were more concerned about whether this feeding method can improve sheep’s health and product quality [7]. For instance, reducing dietary N levels by 50% based on requirements downregulated the cell-mediated immune response and humoral immune response of sheep, as well as adverse effects on production [8]. On the other hand, higher dietary SP levels may be detrimental to ruminant’s immunity and product quality, as high levels of urea supplementation (fully solubility increases SP levels) could reduce nutrient absorption in the rumen [5,9]. However, it is not yet clear what impact SP regulation with decreased CP content in our study will have on sheep’s health and product quality.

Therefore, this study focused on the impact of reducing dietary N levels and regulating SP levels on antioxidant capacity, immunity health and meat quality of sheep based on our previous in vivo experiments. Herein, we hypothesize that oscillating dietary SP levels with decreasing CP can affect antioxidant capacity and inflammatory response to ameliorate meat quality of Hu sheep.

## 2. Materials and Methods

### 2.1. Ethics Statement

In this study, all procedures with animals were approved by the Animal Welfare Committee of Yangzhou Veterinarians, under the Ministry of Agriculture of China (Protocol No. Syxk (Su) 2019-0029).

### 2.2. Dietary Treatments and Animal Management

Thirty-two healthy male Hu sheep (6 months old) were randomly divided into four groups (*n* = 8/group) based on their initial live weight (LW; 40.37 ± 1.18 kg) and individually housed in pens (2.0 m × 1.5 m). They were provided with one of four different diets as follows: The control diet (CON) was formulated according to the nutritional requirements outlined in the National Research Council standard [10] of sheep (LW: 40 kg, average daily gain: 200–250 g/d), with a CP content of 16.7% on a dry matter (DM) basis. The CP content of the other three diets (SPA, SPB and SPC) was reduced by approximately 10% compared to CON, and the proportion of SP was adjusted to 21.2, 25.9 and 29.4%, respectively. All four treatments were isoenergetic, while the SPA, SPB, and SPC diets were isonitrogenous and met 90% of standard requirements (CP content). The target SP levels were mainly achieved by adjusting the addition levels of different types of concentrates, the detection method of dietary protein solubility referred to buffer-soluble N protocols detailed in our previous publication [6].

The sheep were fed twice daily, with the total feed allowance accounting for approximately 3% of LW and maintaining a 50:50 forage-to-concentrate ratio (DM basis), and clean drinking water was provided ad libitum. The experiment lasted for five weeks, including one week of the adaptation period and four weeks of the formal experimental period. Dietary formulations and nutritional compositions are presented in Table 1.

### 2.3. Sample Collection

After fasting overnight and with no access to water for 2 h on the last day of the experiment, sodium thiopental (0.125 mg/kg LW) and 10 mL potassium chloride were administered by intravenous injection to euthanize the sheep before recording LW, and slaughter experiments were subsequently conducted. Blood samples were collected via a vacutainer and serum was harvested after centrifugation (3000× *g*, 4 °C, 15 min). Carcass, muscle (*triceps brachii*, *quadriceps femoris* and *longissimus lumborum*), organs (heart, liver, spleen, lungs, kidneys, and pancreas), the stomachus compositus (SC; including rumen, reticulum, omasum, and abomasum), and intestines were accurately separated after bleeding. Eventually, samples of muscle (*triceps brachii*, *quadriceps femoris* and *longissimus lumborum*), organs (liver, kidneys, and spleen) and digestive tracts (rumen, duodenum, jejunum, and ileum) were acquired (10 g each tissue, washed with cold PBS), promptly frozen in liquid nitrogen and preserved at −80 °C for further analysis. It is worth recalling that the sampling process was completed within 3 min post mortem.

### 2.4. Slaughter Performance and Visceral Organ Indexes

After slaughtering and skinning, the head, tail, limbs, visceral organs, and digestive tracts were removed from each sheep to obtain the carcass, which was weighed for the calculation of the dressing percentage. After the removal of adipose tissue, the bones and meat of the carcass were separated and weighed to estimate meat percentage and bone–meat ratio. Additionally, organs including heart, liver, spleen, lungs, kidneys, and pancreas were thoroughly separated and weighed to calculate organ indexes as percentages of LW. Moreover, SC index (SC weight/LW × 100%) was also determined after the removal of chyme and connective tissue. The rumen, reticulum, omasum, and abomasum were separated and weighed to assess the proportion of each stomach relative to the SC.

### 2.5. Determination of Antioxidant Capacity, Inflammatory Cytokines, and Immunity Responses

The tissue samples were homogenized with a 10% PBS solution (1:9, wt/vol) and the centrifuged to obtain a supernatant for ELISA following a previously established procedure [11]. Antioxidant capacity indexes, including total antioxidants (T-AOC), catalase (CAT), glutathione peroxidase (GSH-Px), superoxide excretion enzyme (SOD), and malondialdehyde (MDA) were assayed using biochemical kits according to the manufacturers’ protocols (Nanjing Jiancheng Bioengineering Institute, Nanjing, China). The concentrations of inflammatory cytokines (IL-1β, IL-6 and IL-8) and immunoglobulin (IgA and IgM) were determined using detection kit instructions (Solarbio Science & Technology Co., Ltd., Beijing, China) via a multifunctional microplate reader (SpectraMax M5; Molecular Devices, Sunnyvale, CA, USA).

### 2.6. Analysis of Meat Quality and Fatty Acids

The carcass was sectioned between the 12th and 13th ribs, and measurements of backfat and rib thickness were taken using vernier calipers (Mitutoyo, Japan) referring to a previous study [12]. Loin muscle area was measured according to established protocol [13]. The pH values of the *triceps brachii*, *quadriceps femoris* and *longissimus lumborum* were measured using a pH meter with a puncture electrode (Testo 205, Lenzkirch, Germany) at 45 min and 24 h post mortem, respectively. The measurement of shear force was performed according to the specified procedure [14]. Briefly, steaks of consistent thickness (approximately 3 cm) were taken from the *triceps brachii*, *quadriceps femoris* and *longissimus lumborum* muscle and then heated in an 80 °C constant temperature water bath. A thermocouple thermometer was used to measure the core temperature of the meat samples, and once the core temperature reached 70 °C, the samples were removed and cooled to a core temperature of 4 °C. Finally, five cores with a diameter of 1.25 cm were cut parallel to the direction of the muscle fibers from the samples and sheared via a tenderness meter (RTA-Meat, Tengba, Shanghai, China), with the blade thickness of 3.0 mm, an inner angle of 60°, a 35 mm height of the inner triangular incision, and 1.00 mm/s of the shear speed. In addition, meat samples from various parts of the carcass were trimmed to a size of 1 cm × 1 cm × 1 cm, with three repetitions for each sample, and weighed after being hung for 24 h at 4 °C to calculate the drip loss rate [15].

Intramuscular fat from the *longissimus lumborum* muscle samples was extracted according to previous protocol [16], and the fatty acid contents were analyzed using a gas chromatograph (Agilent, Wilmington, NC, USA) after esterification. The model of the capillary column (TG-5MS, Thermo, Waltham, MA, USA) was 30 m × 0.25 mm × 0.25 µm (length × diameter × film thickness), and the temperature of the injector and detector was set at 290 °C; the initial temperature of the capillary column was 80 °C, which was maintained for 1 min, then 10 °C/min to 200 °C, 5 °C/min to 225 °C, 2 °C/min to 250 °C, and maintain for 5 min ultimately, with air flow rate and hydrogen flow rate set at 500 mL/min and 50 mL/min, respectively. The fatty acid standard solution and sample determination solution were injected into the gas chromatograph, and then the chromatographic peaks were determined qualitatively and the peak areas were quantitatively measured [17].

### 2.7. Statistical Analysis

Data were analyzed using the one-way ANOVA module in SPSS software (version 16.0) following the following statistical model:

Y_ij_ = μ + T_i_ + S_j_ + e_ij_ (1)

where Y_ij_ is the observation of dependent variables; μ is the overall mean; T_i_ is the fixed effect of treatment; S_j_ is the random sheep effect; and e_ij_ is the residual error for the observation. Multiple comparisons were performed using Tukey’s post hoc test. *p* ≤ 0.05 was considered statistically significant, and a tendency was considered for 0.05 < *p* ≤ 0.10. 

Correlations were calculated using Pearson’s correlation coefficients and Mantel’s test. Heatmap and correlation network maps were visualized using the R packages “ggcor”, “ggplot2”, and “vegan”.

## 3. Results

### 3.1. Slaughter Performance and Organ Indexes

No significant differences were found among treatments in slaughter performance (*p* > 0.05), including LW, carcass weight, dressing percentage, meat percentage, and bone–meat ratio (Table S1). Similarly, there were no significant differences among treatments in the indexes of heart, liver, spleen, lungs, kidneys, and pancreas (*p* > 0.05; Table S2). However, the low-protein treatments resulted in a decreased SC index (*p* < 0.05). Notably, the omasum proportion (% of SC) was increased in SPB compared with other treatments (*p* < 0.05; Table 2).

### 3.2. Antioxidant Capacity

**Figure 1:**
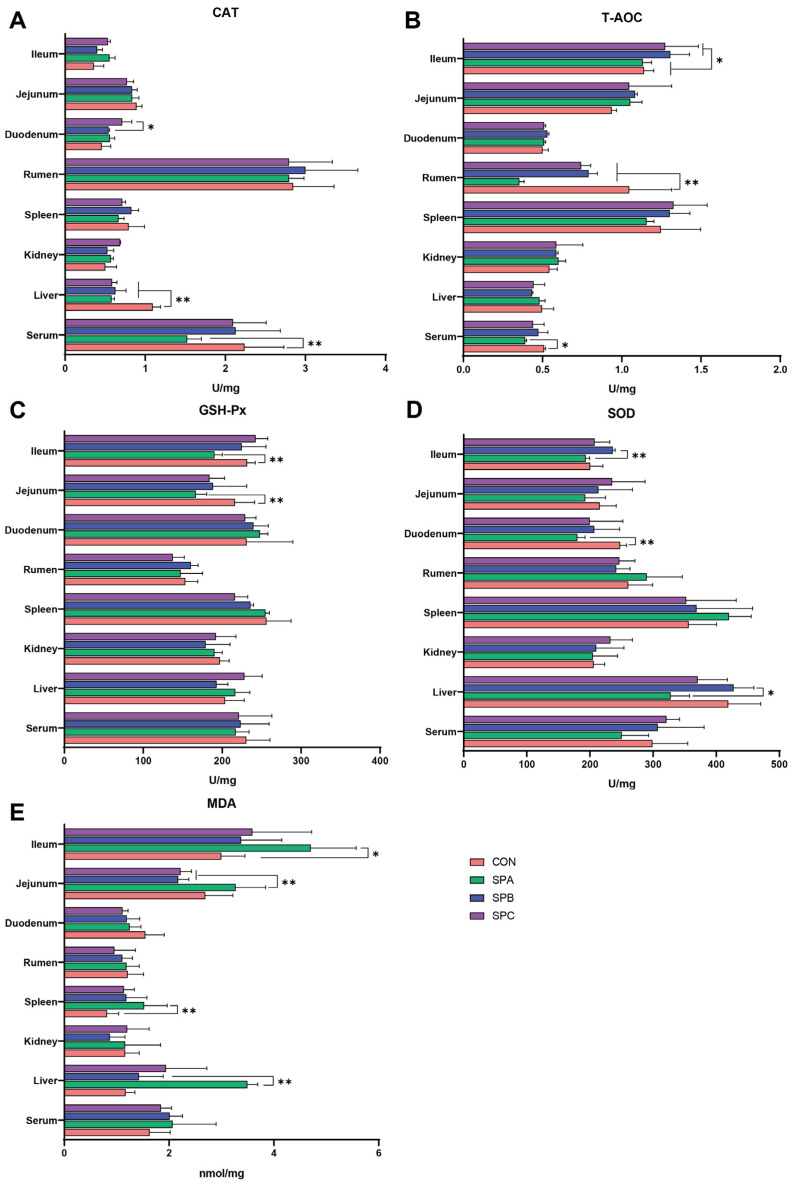
Antioxidant capacity of Hu sheep fed low-protein diets with different soluble protein (SP) proportions. * represents *p* < 0.05 and ** represents *p* < 0.01 between two treatments. (**A**) CAT activity; (**B**) T-AOC; (**C**) GSH-Px activity; (**D**) SOD activity; (**E**) MDA content. CAT = catalase, T-AOC = total antioxidant capacity, GSH-Px = glutathione peroxidase, SOD = superoxide Dismutase, MDA = malondialdehyde. Treatments: CON had a crude protein (CP) content of 16.7% in the diet, based on NRC nutritional requirements; the CP content in the SPA, SPB, and SPC diets was reduced by ~10% with the SP proportion (% of CP) adjusted to 21.2, 25.9, and 29.4%, respectively.

Low-protein treatments led to a reduction in CAT content in the liver (*p* < 0.05; Figure 1A). Meanwhile, CAT content in serum was decreased in SPA compared with CON (*p* < 0.05), and CAT content in the duodenum was higher in SPC than in SPB (*p* < 0.05). The T-AOC content in serum was decreased in the low-protein treatments (*p* < 0.05; Figure 1B), while the T-AOC content in the rumen of SPA was lower than other treatments (*p* < 0.05). Additionally, SPB and SPC increased the T-AOC content in the ileum compared with CON and SPA (*p* < 0.05). Furthermore, the content of GSH-Px in the jejunum and ileum in SPA was lower than those in CON (*p* < 0.05; Figure 1C).

SPB increased SOD content in the liver and ileum compared with SPA (*p* < 0.05; Figure 1D), while the content in the duodenum was lower in SPA than in CON (*p* < 0.05). Moreover, the MDA content in the spleen and ileum was increased in SPA compared with CON (*p* < 0.05; Figure 1E), and the MDA content in the liver was lower in CON and SPB than in SPA (*p* < 0.05). Additionally, SPB and SPC decreased the MDA content in the jejunum compared with SPA (*p* < 0.05).

### 3.3. Pro-Inflammatory Cytokines and Immunity Responses

The content of IL-1β in serum and the rumen increased in SPA compared with CON (*p* < 0.05; Table 3), while the IL-1β content in the spleen of SPA was higher than in CON and SPC (*p* < 0.05). SPA increased the IL-6 content in serum and the duodenum compared with CON (*p* < 0.05), and IL-6 content in the duodenum of SPC was also higher than in CON (*p* < 0.05). Furthermore, SPA increased IL-8 content in serum and the jejunum (*p* < 0.05).

Immunoglobulin contents of different treatments are presented in Table 4. SPB increased IgA content in the serum, liver, and duodenum compared with SPA (*p* < 0.05). On the other hand, IgM content in the liver was lower in SPA than in CON and SPB (*p* < 0.05), while CON obtained a higher IgM content in the duodenum than SPA (*p* < 0.05).

### 3.4. Meat Quality and Fatty Acid Profiles

**Figure 2:**
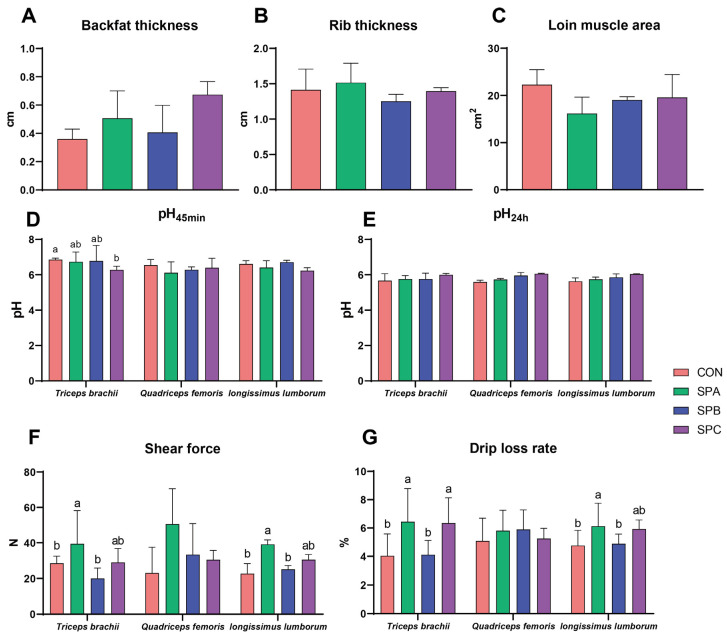
Meat quality of Hu sheep fed low-protein diets with different soluble protein (SP) proportions. (**A**) Backfat thickness; (**B**) Rib thickness; (**C**) Loin muscle area; (**D**) pH_45min_ of different muscles; (**E**) pH_24h_ of different muscles; (**F**) Shear force of different muscles; (**G**) Drip loss rate of different muscles. Bar chart with different superscripts indicates significant differences at *p* < 0.05. Treatments: CON had a crude protein (CP) content of 16.7% in the diet, based on NRC nutritional requirements; the CP content in the SPA, SPB, and SPC diets was reduced by ~10% with the soluble protein proportion (% of CP) adjusted to 21.2, 25.9, and 29.4%, respectively.

No differences were found in backfat thickness, rib thickness, and loin muscle area (*p* > 0.05; Figure 2A–C). However, SPC reduced pH_45min_ in the *triceps brachii* compared with CON (*p* < 0.05; Figure 2D), and there were no differences in pH_24h_ of different tissues among treatments (*p* > 0.05; Figure 2E). Moreover, CON and SPB decreased shear force in the *triceps brachii* and *longissimus lumborum* compared with SPA (*p* < 0.05; Figure 2F). In addition, SPA and SPC increased the drip loss rate in the *triceps brachii* (*p* < 0.05; Figure 2G), while CON and SPB decreased the drip loss rate in the *longissimus lumborum* muscle compared with SPA (*p* < 0.05; Figure 2G).

Regarding the types of fatty acids in the *longissimus lumborum* muscle under different treatments (Table 5), low-protein treatments reduced myristic acid (C14:0) content (*p* < 0.05), and SPB and SPC reduced palmitic acid (C16:0) content compared with CON (*p* < 0.05). In contrast, linoleic acid (C18:2n6c) was increased in SPB and SPC compared with CON (*p* < 0.05), while eicosatrienoic acid (C20:3n3) was upregulated in SPB compared with CON (*p* < 0.05). Furthermore, SPB and SPC decreased saturated fatty acid (SFA) content but increased polyunsaturated fatty acid (PUFA) content compared with CON (*p* < 0.05), mainly reflected in the increase in n-3 PUFA and n-6/n-3 PUFA (*p* < 0.05).

### 3.5. Interactions of Meat Quality of Longissimus Lumborum Muscle with Antioxidant Capacity in Different Organs

**Figure 3:**
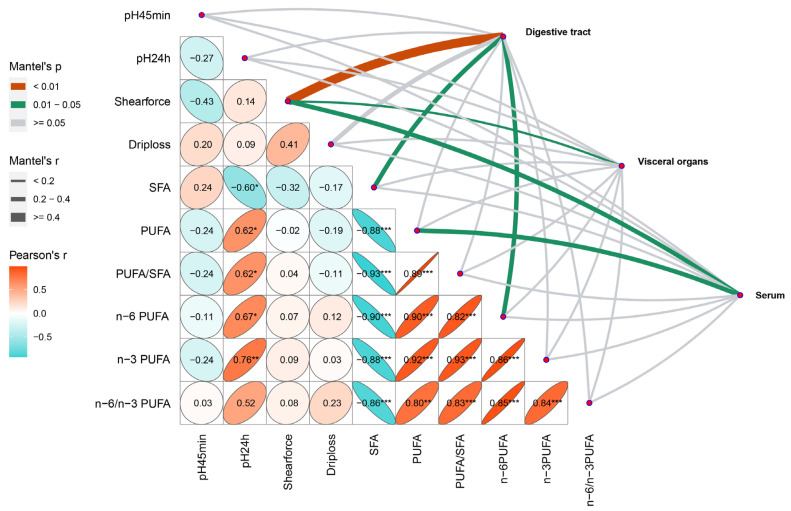
Correlation analysis between antioxidant capacity, including serum, visceral organs (liver/kidney/spleen), and digestive tract (rumen/duodenum/jejunum/ileum) and the meat quality of the *longissimus lumborum* muscle based on Mantel-test coefficient. SFA = saturated fatty acid, PUFA = polyunsaturated fatty acid. * Pearson’s *p* < 0.05, ** Pearson’s *p* < 0.01 and *** Pearson’s *p* < 0.001.

The contents of fatty acid classification were significant correlated with pH value at 24 h post mortem (Pearson’s *p* < 0.05), showing a negative correlation with SFA (Figure 3). On the other hand, PUFA was also negatively correlated with SFA. Moreover, serum antioxidant capacity was correlated with shear force and PUFA (Mantel’s *p* < 0.05). Additionally, visceral organs (liver/kidney/spleen) were also correlated with shear force (Mantel’s *p* < 0.05), while the digestive tracts (rumen/duodenum/jejunum/ileum) were correlated with shear force (Mantel’s *p* < 0.01) and SFA and n-6 PUFA (Mantel’s *p* < 0.05).

## 4. Discussion

Reducing dietary CP levels appropriately would not influence animal production performance. For instance, the previous studies [18] have shown that reducing dietary CP by approximately 30% (from 204 to 132 g/kg) had no discernible effect on the growth and carcass characteristics of young sheep. Additionally, the effect of dietary protein densities on the carcass traits of ram lambs was not as pronounced as that of dietary energy levels [19]. Furthermore, dietary protein levels (125, 140, and 156 g/kg) had no effect on organ indexes of Hu male lambs [20]. Our results align with these previous findings, as we observed no differences in carcass characteristics and organ indexes when CP content decreased by ~10%. Moreover, no difference was observed when adjusting SP levels in low-protein diets. Similarly, it has been reported that carcass characteristics and organ indexes remained unaffected by dietary urea supplementation levels (0, 0.5, 1.5, and 2.5% DM), which were also key to altering dietary SP levels [21]. Interestingly, low-protein diets led to a decrease in the SC index, while the previous studies [22] have shown that feeding oscillating protein diets tended to increase the total stomach weight of calves, which might be attributed to the energy utilization in animals [23]. However, the proportion of omasum (% of SC) was increased in SPB, and the underlying reasons may warrant further investigation.

Accumulation of reactive oxygen species (ROS) may lead to oxidative stress in ruminants, while antioxidant enzymes (e.g., SOD, GSH-Px, and CAT) play a crucial role in mitigating ROS-induced damage to biological macromolecules [24,25]. Antioxidant capacity is closely related to inflammatory response and immunity levels. In this study, the concentrations of CAT and T-AOC in the serum decreased in SPA. Simultaneously, GSH-Px concentration in the small intestine (e.g., jejunum and ileum) also decreased, along with an increase in MDA content. Dietary CP levels affect antioxidant performance, with extremely low or high CP content potentially undermining antioxidant capacity. Optimal antioxidant performance in Huanjiang Mini-Pigs during various growth stages was only achievable within the appropriate CP range [26]. As SP (% of CP) in low-protein diets was adjusted to 21.2%, a decrease in oxidation antioxidant indices and an increase MDA were observed in multiple organs (e.g., serum, liver and small intestine) compared with CON. Our previous results [6] indicated that SPA reduced nutrient digestibility, and an imbalance in nutrient supply at the cellular level could be a contributing factor to the reduced antioxidant capacity. Previous studies [27] have shown that fermented soybean meal (SBM) and enzymatically hydrolyzed SBM can enhance the antioxidant status of weaned piglets. SBM processed through fermentation and enzymatic hydrolysis commonly increased SP content (e.g., small peptides with biological activity), providing advantages over traditional soybean meal in terms of antioxidant capacity [28]. Therefore, it is crucial to maintain an appropriate dietary SP level and select the source of SP to optimize antioxidant status.

The increase in pro-inflammatory cytokines (such as IL-1β, IL-6 and IL-8) signals a potential inflammatory response, which also affects immunity function [29]. In our study, an increase in pro-inflammatory cytokines (IL-1β, IL-6 and IL-8) and a decrease in immunoglobulin (IgA) were observed in the serum of SPA. This could be attributed to the lower antioxidant capacity, as changes in redox signaling play a pivotal role in inflammatory response through the mitogen-activated protein kinase pathway [30]. A study [31] on *Ctenopharyngodon idella* (264.1 ± 0.8 g) suggested that dietary protein content ranging from 287 to 296 g/kg, including 254 to 261 g digestible protein per kg of diet, could inhibit nuclear translocation of NF-κB in fish gills, reducing pro-inflammatory cytokine production (e.g., IL-1β, IL-8 and TNF-α). Similar to the previous section discussing the role of fermented SMB in antioxidant capacity, feeding broiler chicken fermented SMB increased IgM and IgA without altering IgG in the blood [32]. Furthermore, feeding fermented SBM, instead of SBM, reduced the concentration of pro-inflammatory mediators resulting from weaning stress in calves [33]. Notably, in our study, SPA (21.2% SP) increased the serum contents of pro-inflammatory cytokine (IL-1β, IL-6 and IL-8) and decreased the immunoglobulin contents (IgA and IgM) in the liver and duodenum. As SP proportions increased (25.9% and 29.4%) in low-protein diets, the inflammatory response appeared to improve. It was reported [34] the adjusting the content of rapid degradation protein to an appropriate range could maintain the synchronization of rumen carbon and N degradation and ultimately enhance nutrient utilization efficiency and improve immunity response.

Dietary protein levels can affect the meat quality of sheep. For example, [35], low-protein (LP) diets reduced the muscle fiber diameter but increased the muscle fiber density of the *longissimus lumborum* in Tibetan sheep, which may affect muscle tenderness. On the other hand, LP diets decreased the total amino acids and unsaturated fatty acid (UFAs) and increased saturated fatty acid (SFAs) in Hu lamb meat [20]. In the present experiment, reducing CP by 10% had no apparent impact on meat quality. However, as SP levels increased, shear force and drip loss rate of SPB decreased compared with SPA in the *triceps brachii* and *longissimus lumborum* muscle. Similar to previous research results [21], 2.5% urea supplementation in the diet increased muscular pH at 24 h post-slaughter compared with 0% urea, and the meat shear force of diets with 0.5% urea addition was lower than in diets with 0% and 2.5% urea. It is worth mentioning that SPB and SPC contained 0.2% and 0.5% urea, respectively, which was also a core element in the manipulation of SP. Although the proportion of urea added was very low, we speculate that SP levels (% of CP) of 25.9% and 29.4% might be relatively conservative proportions, and our results would provide a lower limit for SP level under the dietary pattern. 

Dietary nutrition intervention is one of the primary factors affecting the distribution of fatty acids in ruminant muscle, and innovative nutritional strategies can achieve healthy fatty acids in lamb meat [36]. Our remarkable finding is that regulating SP (% of CP) to 25.9% and 29.4% and reducing CP by ~10% decreased SFA content and increased PUFA content (especially n-3 PUFA) in the *longissimus lumborum* muscle. Some studies have shown that SFAs may be associated with health issues such as arteriosclerosis and cardiovascular disease, while PUFAs may enhance the texture, aroma and flavor of meat, which is also beneficial for human health and well-being, particularly n-3 PUFA [37,38]. Similar to our results, feeding broiler partial replacement of fermented rapeseed cake for SBM reduced SFA content and increased UFA content, while a low proportion of n-6/n-3 PUFA in breast muscle was observed [39]. It has been demonstrated that choice of SP source and its levels (such as the appropriate increase in bioactive peptides and amino acids) can enhance meat quality and safety [40].

Finally, we explored the interactions between the meat quality of the *longissimus lumborum* muscle and antioxidant capacity in different organs. We found that the antioxidant capacity of all organs we tested in this study was related to shear force, with the degree of correlation ranked from digestive tract, serum, to visceral organs according to the Mantel test (*p*-value from low to high). This indicated the importance of body antioxidant capacity on meat tenderness [41]. It is also reported that dietary supplementation with vitamin E improved the oxidative stability of pork lipids and reduced drip loss, as well as enhanced tenderness [42]. The antioxidant capacity of the digestive tract appeared to be the primary factor affecting meat quality in our results, and it was also correlated with SFA and n-6 PUFA. Rumen, duodenum, jejunum, and ileum are crucial for nutrient absorption and transport in ruminants, and the decreased antioxidant capacity in these organs may be linked to an imbalance in nutrient supply, such as the failure of simultaneous energy and N release in the rumen [43]. Therefore, our results highlight the importance of maintaining appropriate protein solubility in the diet for the body’s antioxidant capacity and meat quality, offering a reference for the lower threshold of dietary SP levels.

## 5. Conclusions

Although regulating dietary SP levels (% of CP) from 21.2% to 29.4% with decreased CP content reduced the SC index, it did not affect slaughter performance. Lower dietary SP levels (21.2% of CP) would lead to a decrease in oxidation antioxidant indices and an increase in MDA and inflammatory cytokines in multiple organs (e.g., serum and small intestine), resulting in a decrease in meat quality, such as increased shear force and SFA content, as well as reduced water-holding capacity and PUFA content of the *longissimus lumborum* muscle. However, the situation has improved as SP proportion increased to 25.9 and 29.4%; meanwhile the highest correlation of antioxidant capacity of digestive tracts (rumen, duodenum, jejunum, and ileum) with meat quality also underscored the importance of maintaining a balanced nutrient absorption (e.g., simultaneous release of energy and N). Finally, it is worth noting that the choice of SP sources, which contain more bioactive peptides and amino acids as SP beneficial to physiology, should be considered alongside the maintenance of appropriate SP levels.

## Figures and Tables

**Table 1:** Ingredients and nutritive levels of the experimental diets.

Items	Treatments			
	CON	SPA	SPB	SPC
Ingredient, % of DM				
Mixed silage ^1^	50	50	50	50
Corn	34	35.5	35.65	38
Soybean meal	5	-	1.5	-
Wheat bran	8.5	11	11	10
Urea	-	-	0.2	0.5
Corn protein meal	1	2	0.15	-
NaHCO_3_	0.5	0.5	0.5	0.5
Premix ^2^	0.5	0.5	0.5	0.5
NaCl	0.5	0.5	0.5	0.5
Total	100	100	100	100
Nutritive level, g/kg (measured values except as noted)				
DE (MJ/kg) ^3^	14.73	14.65	14.81	14.76
CP	166.9	155.1	155.7	156.5
SP (% of CP)	23.5	21.2	25.9	29.4
EE	33.5	34.6	34.5	34.8
NDF	459.9	442.1	471.2	478.7
ADF	271.7	266.7	274.9	276.2
Ash	123.3	123.2	119.6	115.6
Ca	4.6	4.4	4.5	4.5
P	3.9	4.2	4.2	4.4

^1^ Cabbage and straw were combined in a silage mixture with a ratio of 6:4 (DM basis), with the inclusion of 0.035 g/kg *Lactobacillus plantarum* and 0.250 g/kg cellulase. ^2^ The formulated composition, per kilogram of DM, included vitamin A (130 KIU), vitamin D_3_ (30 KIU), vitamin E (13 KIU), Fe (0.8 g), Mn (1.0 g), Zn (3.0 g), Cu (0.2 g), Se (8 mg), and NaCl (100 g). ^3^ Calculated according to the nutrient requirements for fattening sheep. DM = dry matter; DE = digestible energy; CP = crude protein; SP = soluble protein; EE = ether extract; NDF = neutral detergent fiber; ADF = acid detergent fiber; Ca = calcium; P = phosphorus. Treatments: CON had a crude protein (CP) content of 16.7% in the diet, based on NRC nutritional requirements; the CP content in the SPA, SPB, and SPC diets was reduced by ~10% with the soluble protein proportion (% of CP) adjusted to 21.2, 25.9, and 29.4%, respectively. This table is cited from Zhang et al. (2022) [6].

**Table 2:** Development of stomachus compositus (SC) of Hu sheep under different treatments.

Items	Treatments				SEM	*p*-Value
	CON	SPA	SPB	SPC		
SC, kg	1.24	1.02	0.93	0.97	0.191	0.225
SC Index, %	2.67 ^a^	2.19 ^b^	1.96 ^b^	2.12 ^b^	0.365	0.046
Rumen, % of SC	64.87	61.36	57.68	62.79	4.273	0.213
Reticulum, % of SC	9.17	12.26	13.03	10.62	2.652	0.309
Omasum, % of SC	9.39 ^b^	9.71 ^b^	14.17 ^a^	8.87 ^b^	2.911	0.032
Abomasum, % of SC	16.56	16.68	15.13	17.72	2.437	0.692

SC Index = SC weight/live weight × 100%; SEM = standard error of the mean. Treatments: CON had a crude protein (CP) content of 16.7% in the diet, based on NRC nutritional requirements; the CP content in the SPA, SPB, and SPC diets was reduced by ~10% with the soluble protein proportion (% of CP) adjusted to 21.2, 25.9, and 29.4%, respectively. Means with different superscripts were significantly different (*p* < 0.05).

**Table 3:** Concentrations of pro-inflammatory cytokines in Hu sheep under different treatments.

Parameters	Organ Type	Treatments				SEM	*p*-Value
		CON	SPA	SPB	SPC		
IL-1β (pg/mL)	Serum	363.51 ^b^	589.87 ^a^	499.68 ^ab^	467.19 ^ab^	54.629	0.001
	Liver	367.66	541.42	415.55	512.15	36.913	0.089
	Kidney	451.63	562.06	415.54	465.16	36.893	0.112
	Spleen	494.15 ^b^	654.68 ^a^	554.61 ^ab^	455.21 ^b^	59.141	0.044
	Rumen	405.88 ^b^	654.61 ^a^	455.21 ^ab^	456.46 ^ab^	73.556	0.012
	Duodenum	483.07	738.63	575.25	604.47	67.917	0.123
	Jejunum	583.30	559.52	451.61	654.51	50.263	0.434
	Ileum	302.57	582.50	496.86	413.90	18.802	0.323
IL-6 (pg/mL)	Serum	147.88 ^b^	279.06 ^a^	235.61 ^ab^	205.29 ^b^	21.408	0.001
	Liver	389.78	456.41	365.98	489.71	30.001	0.367
	Kidney	254.22	294.63	246.54	286.40	33.904	0.372
	Spleen	145.45	154.46	125.46	142.14	67.911	0.579
	Rumen	162.45	154.22	142.15	148.26	35.267	0.635
	Duodenum	396.98 ^b^	460.92 ^a^	408.55 ^ab^	493.55 ^a^	68.661	0.028
	Jejunum	367.19	401.07	322.97	382.59	92.674	0.147
	Ileum	320.79	348.85	308.64	333.74	46.670	0.211
IL-8 (pg/mL)	Serum	12.72 ^b^	13.67 ^a^	12.20 ^b^	12.62 ^ab^	1.689	0.002
	Liver	8.41	9.62	9.65	10.65	0.933	0.621
	Kidney	6.55	6.57	5.97	6.65	0.699	0.517
	Spleen	4.65	4.55	4.11	5.15	1.184	0.575
	Rumen	2.26	2.32	2.21	3.51	0.917	0.804
	Duodenum	4.75	6.38	5.71	6.07	0.488	0.358
	Jejunum	4.31 ^b^	5.91 ^a^	4.16 ^b^	4.02 ^b^	0.376	0.043
	Ileum	5.20	5.30	4.26	4.85	0.183	0.186

IL-1β = interleukin-1β, IL-6 = interleukin-6, IL-8 = interleukin-8. SEM = standard error of the mean. Treatments: CON had a crude protein (CP) content of 16.7% in the diet, based on NRC nutritional requirements; the CP content in the SPA, SPB, and SPC diets was reduced by ~10% with the soluble protein proportion (% of CP) adjusted to 21.2, 25.9, and 29.4%, respectively. Means with different superscripts were significantly different (*p* < 0.05).

**Table 4:** Immunoglobulin contents in Hu sheep under different treatments.

Items	Organ Type	Treatments				SEM	*p*-Value
		CON	SPA	SPB	SPC		
IgA (ug/mL)	Serum	24.30 ^b^	24.36 ^b^	30.02 ^a^	28.15 ^ab^	8.611	0.001
	Liver	56.42 ^a^	45.29 ^b^	54.22 ^a^	49.16 ^ab^	3.668	0.033
	Kidney	31.54	26.51	41.51	42.62	4.829	0.089
	Spleen	21.54	24.21	22.54	25.16	2.997	0.572
	Rumen	57.84	65.42	59.42	68.98	3.825	0.093
	Duodenum	105.98 ^a^	77.31 ^b^	100.01 ^a^	91.80 ^ab^	8.655	0.015
	Jejunum	54.51	46.06	52.02	51.76	2.774	0.301
	Ileum	68.72	55.80	65.41	56.06	11.239	0.638
IgM (ug/mL)	Serum	400.55	368.54	377.91	396.00	60.713	0.093
	Liver	351.27 ^a^	314.65 ^b^	364.13 ^a^	346.13 ^ab^	82.516	0.035
	Kidney	145.13	142.11	146.51	141.42	40.373	0.452
	Spleen	214.65	214.56	146.52	261.45	59.537	0.301
	Rumen	717.56	704.52	715.67	714.56	55.175	0.497
	Duodenum	734.92 ^a^	620.77 ^b^	725.39 ^ab^	729.36 ^ab^	56.642	0.034
	Jejunum	458.15	410.13	414.76	452.68	58.993	0.638
	Ileum	610.13	584.45	643.55	663.68	77.570	0.044

IgA = immunoglobulin A, IgM= immunoglobulin M. SEM = standard error of the mean. Treatments: CON had a crude protein (CP) content of 16.7% in the diet, based on NRC nutritional requirements; the CP content in the SPA, SPB, and SPC diets was reduced by ~10% with the soluble protein proportion (% of CP) adjusted to 21.2, 25.9, and 29.4%, respectively. Means with different superscripts were significantly different (*p* < 0.05).

**Table 5:** Fatty acid contents (%) in the *longissimus lumborum* muscle of Hu sheep under different treatments.

Parameters	Treatments				SEM	*p*-Value
	CON	SPA	SPB	SPC		
Fatty acid composition						
C10:0	0.11	0.16	0.12	0.09	0.004	0.062
C12:0	0.03	0.05	0.05	0.06	0.001	0.105
C13:0	0.70	0.59	0.71	0.66	0.021	0.211
C14:0	5.91 ^a^	2.52 ^b^	2.25 ^b^	2.32 ^b^	0.256	0.015
C14:1	0.08	0.11	0.10	0.13	0.009	0.554
C15:0	0.36	0.23	0.25	0.19	0.022	0.578
C15:1	17.56	19.80	20.59	18.99	1.156	0.118
C16:0	27.80 ^a^	26.01 ^ab^	24.15 ^b^	25.43 ^b^	2.189	0.034
C16:1	1.15	1.02	0.98	1.12	0.054	0.582
C17:0	0.77	0.80	0.86	0.88	0.044	0.185
C18:0	1.51	1.23	0.97	1.12	0.106	0.755
C18:1n9c	0.72	0.52	0.44	0.61	0.032	0.102
C18:2n6c	3.35 ^b^	4.01 ^ab^	4.89 ^a^	4.77 ^a^	0.258	0.044
C20:0	0.06	0.04	0.08	0.05	0.005	0.199
C18:3n6	0.05	0.03	0.09	0.06	0.003	0.103
C18:3n3	0.11	0.09	0.12	0.18	0.008	0.733
C20:1	0.03	0.02	0.06	0.04	0.001	0.383
C20:2	0.09	0.08	0.06	0.08	0.002	0.884
C20:3n3	2.56 ^b^	2.81 ^ab^	3.16 ^a^	3.02 ^ab^	0.156	0.022
C22:1n9	0.81	0.9	1.01	0.99	0.083	0.382
C23:0	0.21	0.32	0.36	0.29	0.004	0.229
C22:6n3	0.14	0.16	0.22	0.18	0.003	0.244
Fatty acid classification						
SFA	55.1 ^a^	51.86 ^ab^	50.49 ^b^	50.21 ^b^	4.532	0.003
MUFA	20.35	22.37	23.18	21.88	2.835	0.221
PUFA	6.30 ^b^	7.18 ^ab^	8.54 ^a^	8.29 ^a^	0.582	0.034
PUFA/SFA	0.11	0.14	0.17	0.17	0.001	0.332
n-6 PUFA	3.4	4.04	4.98	4.83	0.224	0.144
n-3 PUFA	2.81 ^b^	3.06 ^ab^	3.50 ^a^	3.38 ^a^	0.002	0.025
n-6/n-3 PUFA	1.21 ^b^	1.32 ^ab^	1.42 ^a^	1.43 ^a^	1.976	0.034

SFA = saturated fatty acid, MCFA = medium-chain fatty acid, MUFA = monounsaturated fatty acid, PUFA = polyunsaturated fatty acid. SEM = standard error of the mean. Treatments: CON had a crude protein (CP) content of 16.7% in the diet, based on NRC nutritional requirements; the CP content in the SPA, SPB, and SPC diets was reduced by ~10% with the soluble protein proportion (% of CP) adjusted to 21.2, 25.9, and 29.4%, respectively. Means with different superscripts were significantly different (*p* < 0.05).

## Data Availability

Data are contained within the article and Supplementary Materials.

## Supplementary Materials

The following are available online at https://www.mdpi.com/article/10.3390/antiox12122098/s1, Table S1: Slaughter performance of Hu sheep fed low-protein diets with different soluble protein (SP) proportions. Table S2: Visceral organs index (%) of Hu sheep under different treatments.
