# Supplementary material for: Antioxidant Capacity, Inflammatory Response, Carcass Characteristics and Meat Quality of Hu Sheep in Response to Dietary Soluble Protein Levels with Decreased Crude Protein Content
Source: Antioxidants (Basel). 2023 Dec 11;12(12):2098. doi: 10.3390/antiox12122098 (PMC10741046; doi:10.3390/antiox12122098)
Supplement: Supplementary file 1 [file antioxidants-12-02098-s001.zip › antioxidants-2710406-supplementary.pdf]

Table S1. Slaughter performance of Hu sheep fed low-protein diets with different soluble protein (SP) proportions.

Parameters	Treatments				SEM	*P*-Value
	CON	SPA	SPB	SPC		
Live weight, kg	46.41	46.59	47.42	45.72	0.753	0.527
Carcass weight, kg	20.42	21.21	21.22	20.78	0.701	0.747
Dressing percentage, %	43.99	45.53	44.74	45.45	0.653	0.228
Meat percentage, %	31.16	31.66	33.26	32.32	0.744	0.354
Bone–meat ratio	0.53	0.51	0.46	0.48	0.021	0.312

SEM = standard error of the mean. Treatments: CON had a crude protein (CP) content of 16.7% in the diet, based on NRC nutritional requirements; the CP content in the SPA, SPB, and SPC diets was reduced by ~10% with the SP proportion (% of CP) adjusted to 21.2, 25.9, and 29.4%, respectively.

Table S2. Visceral organs index (%) of Hu sheep under different treatments.

Items	Treatments				SEM	*P*-Value
	CON	SPA	SPB	SPC		
Heart	0.43	0.38	0.38	0.40	0.052	0.566
Liver	1.62	1.43	1.47	1.45	0.209	0.680
Spleen	0.16	0.17	0.18	0.19	0.043	0.866
Lung	0.96	0.95	1.12	1.03	0.312	0.932
Kidney	0.27	0.22	0.24	0.26	0.055	0.692
Pancreas	0.35	0.41	0.48	0.35	0.097	0.245

SEM = standard error of the mean. Treatments: CON had a crude protein (CP) content of 16.7% in the diet, based on NRC nutritional requirements; the CP content in the SPA, SPB, and SPC diets was reduced by ~10% with the soluble protein proportion (% of CP) adjusted to 21.2, 25.9, and 29.4%, respectively.
